# Antibiotic-loaded spacer arthroplasty in a terrible triad injury with unreconstructable radial head fracture: a case report

**DOI:** 10.1186/s13256-023-04258-5

**Published:** 2023-12-08

**Authors:** Hasan Barati, Mojtaba Baroutkoub, Nikaein Zabeti Targhi, Shahabaldin Beheshti Fard, Bardia Hajikarimloo, Sina Afzal

**Affiliations:** 1 Department of Orthopedic Surgery, School of Medicine, Shahid Beheshti University of Medical Sciences, Tehran, Iran; 2 Student Research Committee, School of Medicine, Shahid Beheshti University of Medical Sciences, Tehran, Iran; 3 Joint Reconstruction Research Center (JRRC), Tehran University of Medical Sciences, Tehran, Iran

**Keywords:** Terrible triad, Radial head, Spacer, Arthroplasty, Elbow, Case report

## Abstract

**Background:**

Radial head arthroplasty is a viable option in cases with terrible triad injuries of elbow, wherein the radial head sustains significant comminution that precludes reconstruction. Nevertheless, this alternative is not recommended for individuals with poor elbow skin conditions, accompanied neuropsychiatric disorders, or low patient compliance. This case report presents a patient with bilateral terrible triad injury, along with the aforementioned conditions. The report outlines the treatment challenges of such a case and proposes potential solutions.

**Case presentation:**

A 37-year-old Persian male patient presenting with a bilateral terrible triad fracture–dislocation and a history of psychoactive substance abuse, was admitted to our emergency department. The patient underwent radial head replacement using a cement spacer containing antibiotics, due to the comminuted radial head in the presence of a contaminated wound on the left elbow. The fracture of the right side was successfully fixed. Subsequent to discharge, the patient did not attend any follow-up appointments. After a period of 6 months, he was admitted to the psychiatric ward and orthopedic consultation was requested to evaluate the patient.

**Conclusion:**

In acute terrible triad injuries with unreconstructable radial head fractures where arthroplasty with metallic prostheses may not be suitable due to contaminated wounds, unstable psychiatric condition, and low patient cooperation, temporary orthopedic cement spacers can maintain elbow biomechanics, stability, and sterility.

## Introduction

The terrible triad injury (TTI) of the elbow is defined as posterior elbow dislocation in conjunction with fractures of the coronoid process and radial head (RH). Hotchkiss introduced this concept to the literature in 1996 [1]. As the name implies, this condition is associated with poor clinical outcomes, so the management of these injuries has always been a challenge for orthopedic surgeons. In case of TTI occurring in both elbows, the patient would experience a significant decline in quality of life due to severity of this injury [2].

Except for limited cases, surgical intervention is necessary to attain a stable and congruent reduction in TTIs. When the RH fracture is so comminuted that fixation is impossible, then RH should be replaced, because resection is contraindicated in TTIs. The coexistence of a contaminated wound and an unreconstructable RH fracture poses a significant challenge in determining the optimal surgical option, since arthroplasty is contraindicated in cases with poor skin quality of the elbow [3].

The purpose of this article is to present a case with bilateral elbow TTI in an individual with psychiatric issues, a contaminated large abrasion, and a comminuted RH fracture, which is an exceedingly uncommon co-occurrence. Furthermore, we discuss the treatment complexities associated with this specific condition and present our proposed approach. On the basis of our review of the literature, no instances of a comparable terrible triad injury involving the aforementioned challenges have been identified.

## Case presentation

### Patient history and presentation

**Fig. 1:**
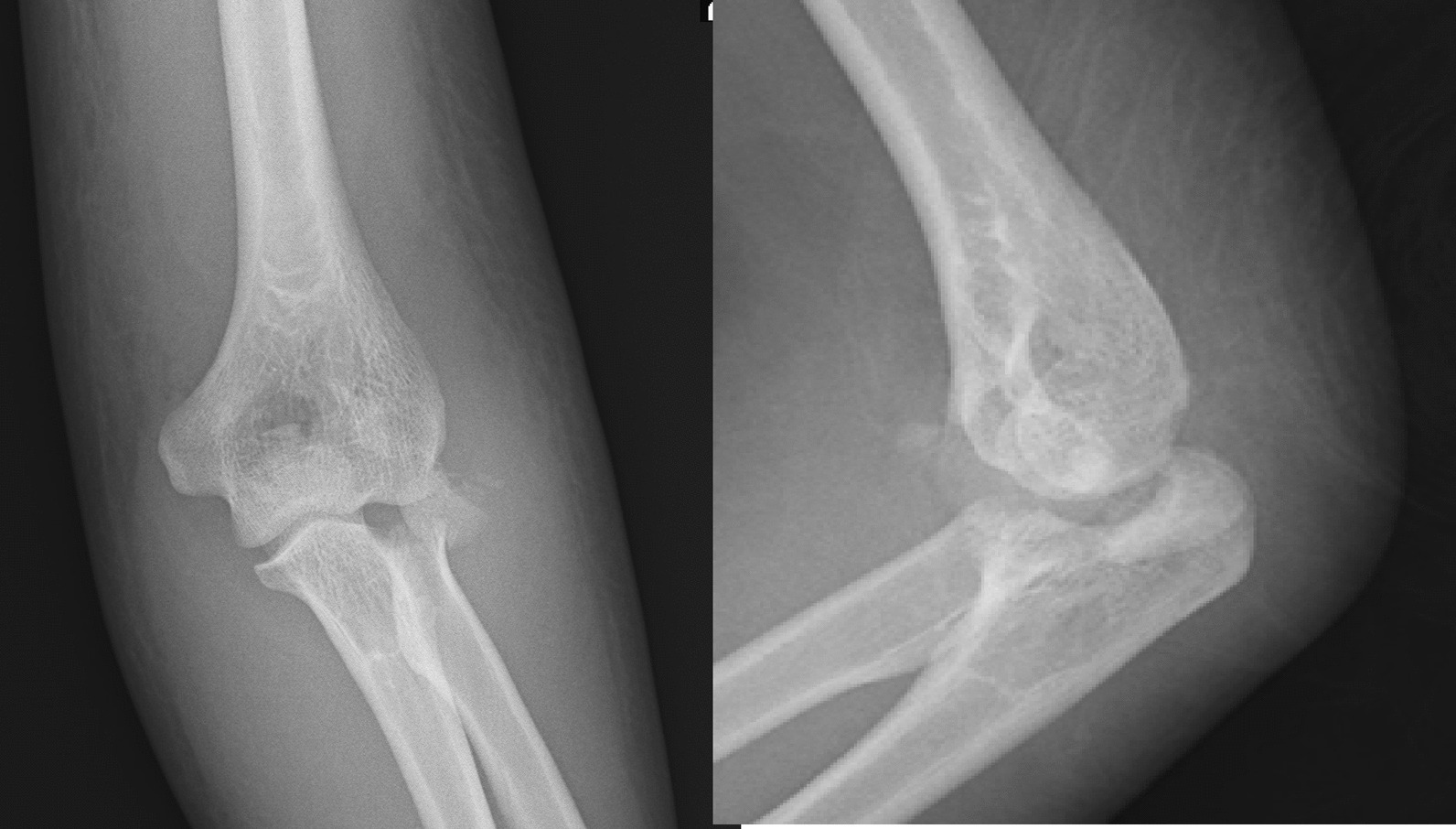
Post-reduction anteroposterior and lateral view X-ray of the left elbow; the anteroposterior view reveals the presence of a displaced radial head fracture, while the lateral view indicates the occurrence of a coronoid fracture

**Fig. 2:**
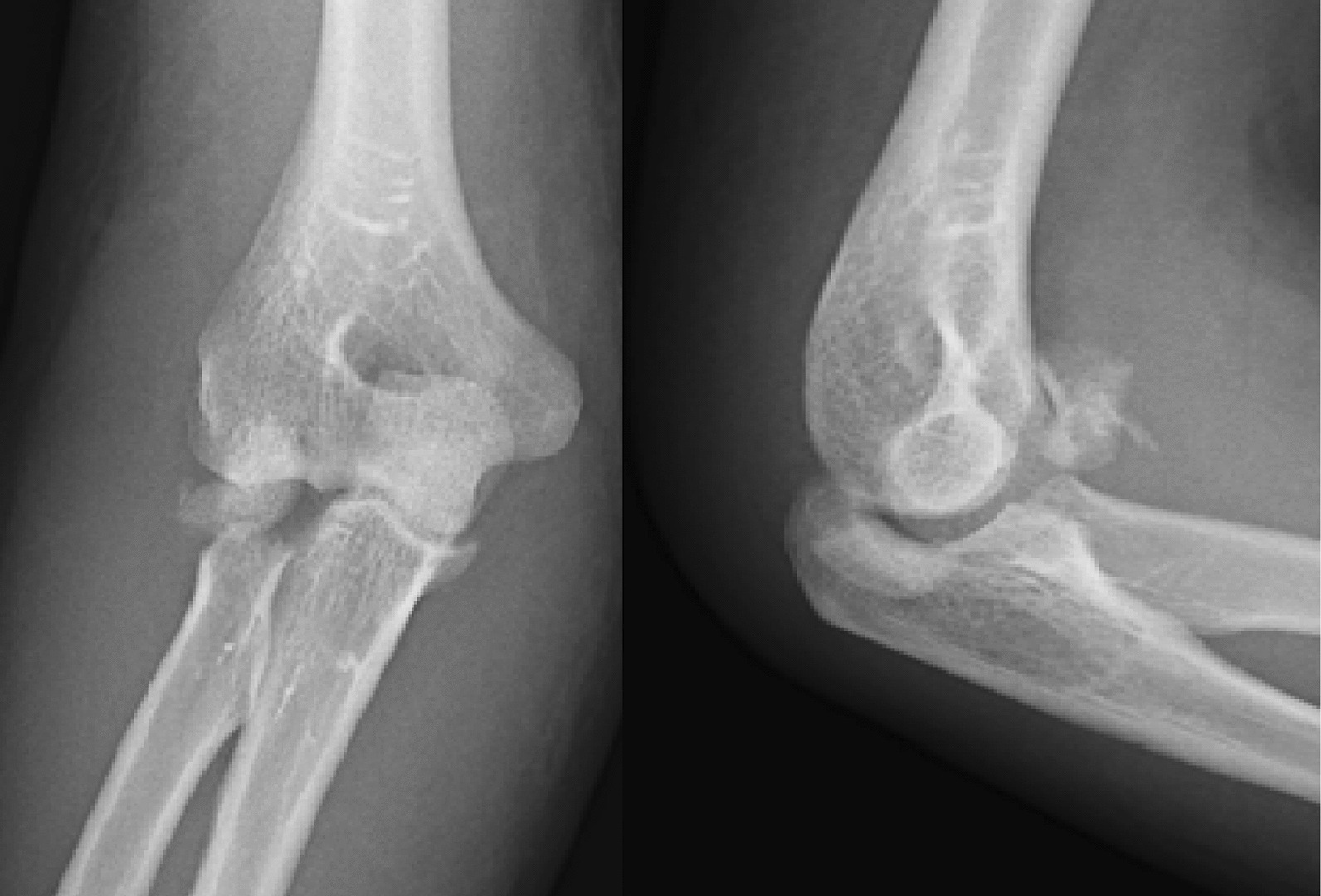
Post-reduction anteroposterior and lateral view X-ray of the right elbow

**Fig. 3:**
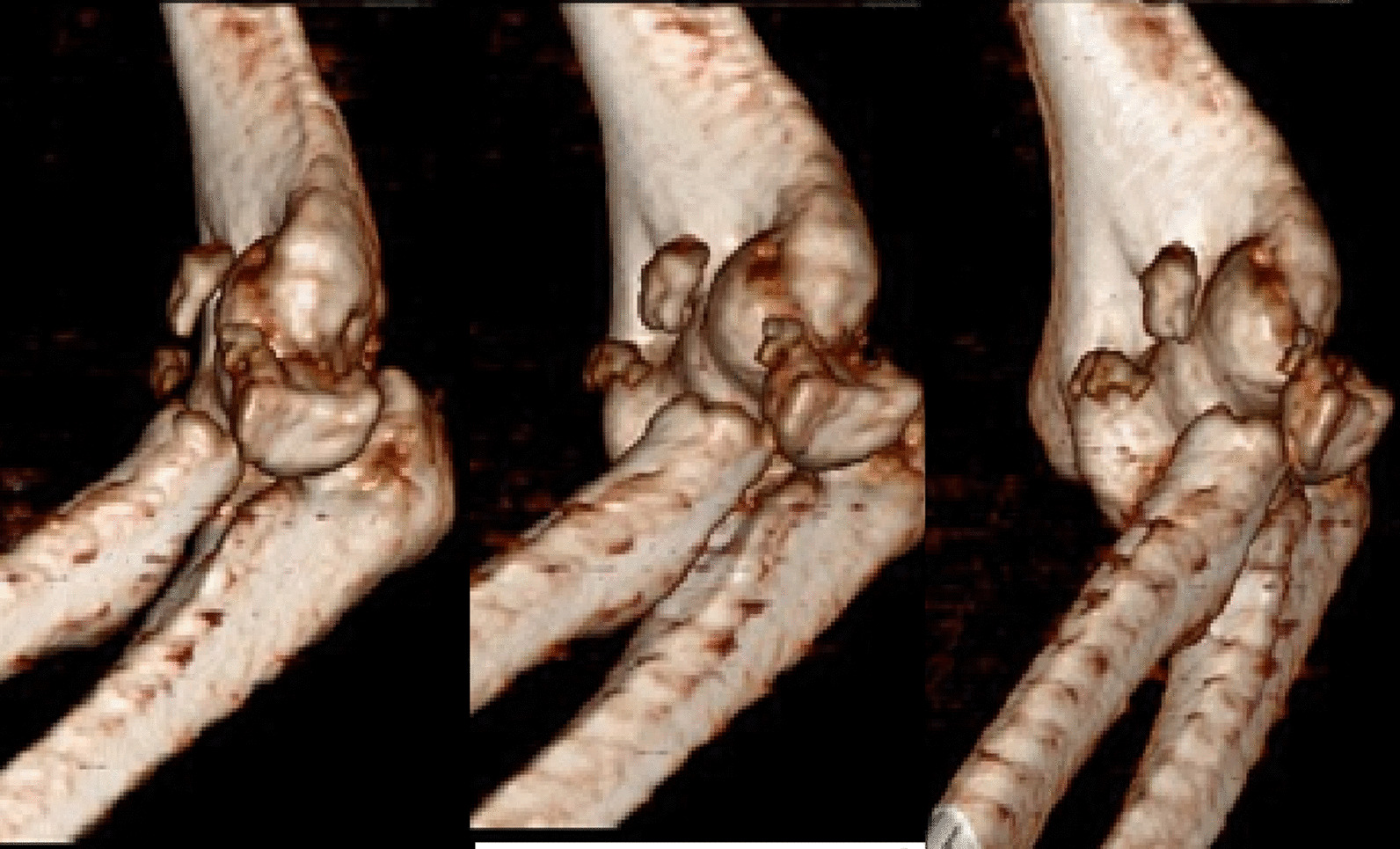
Left elbow computed tomography (CT) imaging; radial head comminution (Mason type 4) and coronoid fracture (Regan–Morris type 1) are described in great depth

**Fig. 4:**
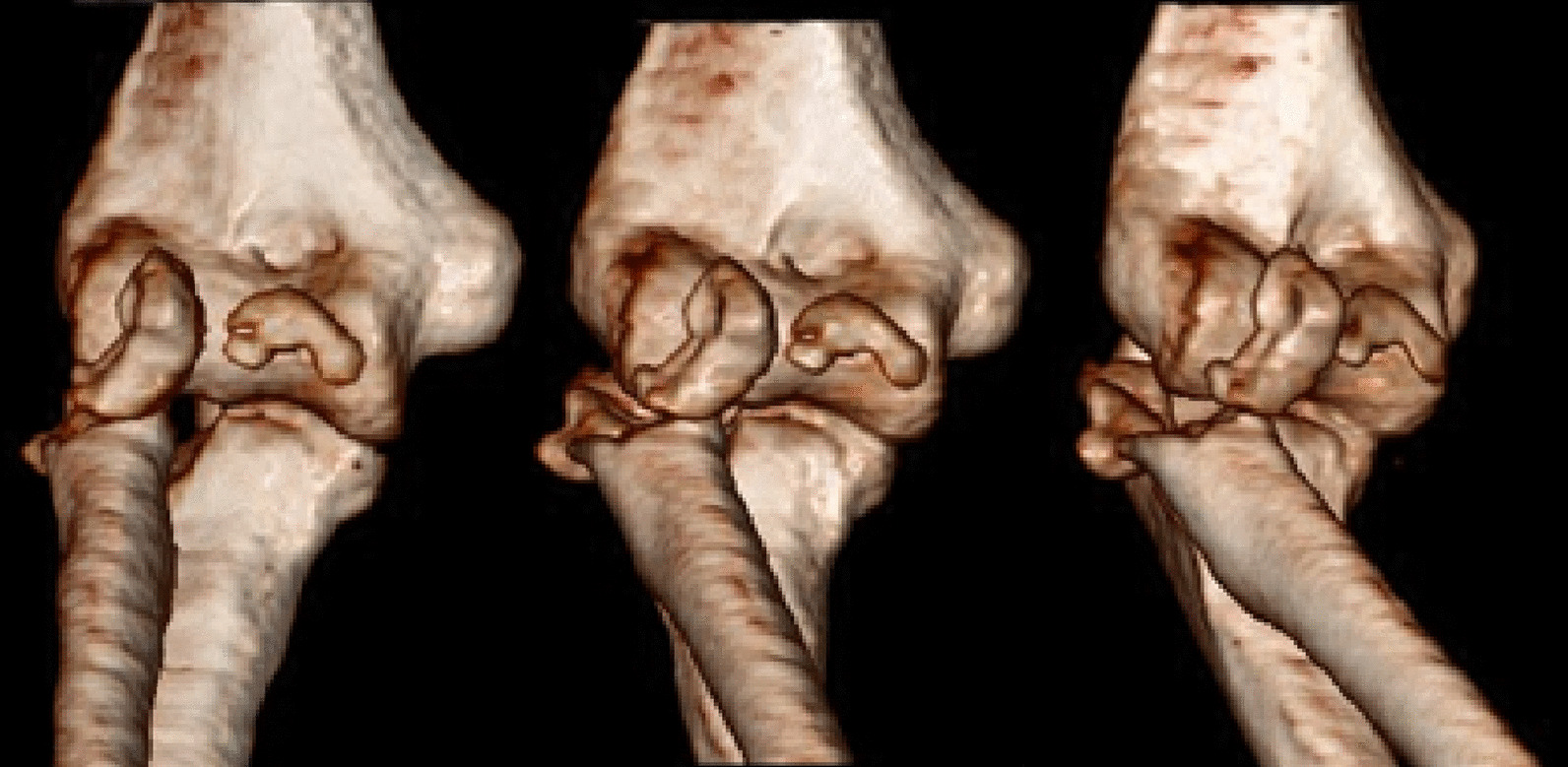
Right elbow computed tomography (CT) imaging; radial head fracture (Mason type 4) and coronoid fracture (Regan–Morris type 2) are described in great depth

A 37-year-old Persian man was referred to our tertiary hospital with bilateral TTIs following a fall from a height of 3 m. Both elbows were reduced at the primary center. A contaminated deep abrasion measuring approximately 5 × 3 cm was observed on the lateral proximal of the left forearm. Vascular and neurological examinations of both upper extremities were normal. Plain radiographs were obtained and evaluations demonstrated subluxations of both elbows associated with RH fracture (Mason type 4) [4] and coronoid process fracture (Regan–Morrey type 2) [5] (Figs. 1,2). The thorough clinical, imaging, and laboratory assessments revealed no signs of other associated trauma elsewhere. Apart from psychiatric disorders resulting from the use of psychoactive substances, the patient’s medical history was unremarkable. Three-dimensional computed tomography (3D-CT) scan exhibited features of the TTI in more detail (Figs. 3,4).

### Surgical treatment

Following preoperative measures, including psychiatric consultation, the patient underwent surgery performed by a team of two surgeons within 48 hours of the trauma event. In the supine position and under general anesthesia, after applying the tourniquets, first the patient’s wound was irrigated and debrided. Subsequently, the RH fragments were bilaterally approached via the Kocher interval [6], and we tried to reconstruct the RHs like pieces of a puzzle. The coronoid fracture and anterior joint capsule rupture in the left elbow underwent reduction and repair via the utilization of a transosseous tunnel, while the right elbow underwent the same procedure using an anchor suture. All of these procedures were carried out with the forearm pronated to limit the risk of injury to the posterior interosseous nerve.

**Fig. 5:**
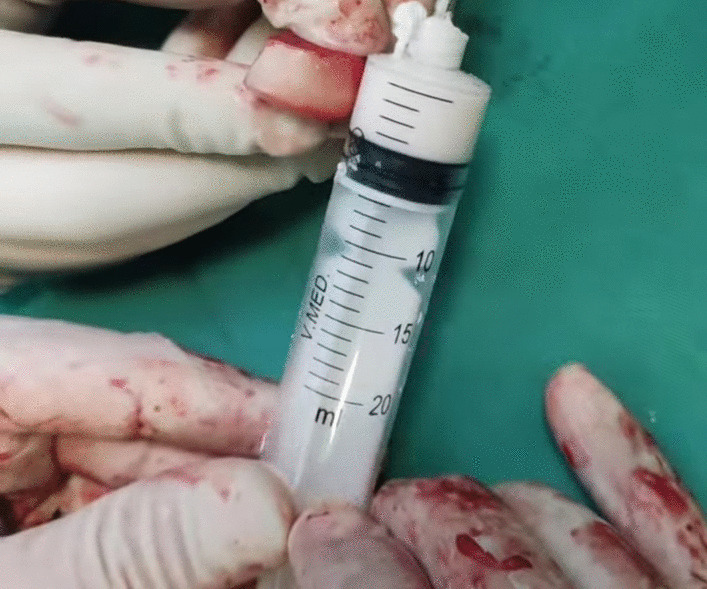
Preparation of radial head spacer; image depicts the process of determining the spacer height by utilizing the resected radial head

Following the successful reconstruction of right RH, the head was fixed to the radial neck using a mini plate. However, in the left side, due to the severe comminution, reconstruction was not possible, and the head was replaced with a temporary antibiotic-impregnated spacer. To make an antibiotic spacer, we mixed a pack of orthopedic cement of polymethyl methacrylate (PMMA) with 3.6 g of tobramycin and 1 g of vancomycin. Subsequently, the approximate diameter of resected head was measured, which was greater than the inner diameter of the 20 cc syringe and smaller than the 30 cc syringe. Consequently, using the smaller 20 cc syringe, a molded spacer was made with a height equivalent to that of the excised head, along with a stem fashioned from a Kirschner wire coated with cement (Fig. 5).

After the surgical treatment of the bilateral coronoid and RH injuries, the lateral collateral ligaments (LCL) on both sides, which were torn from their origin of the humeral lateral epicondyles, were repaired using transosseous tunnels. The sutures were tightened while the forearm was pronated and the elbow was at 90 degrees of flexion. The stability of the elbow joint under valgus stress was then checked via the fluoroscopy guide, which indicated intact medial collateral ligaments (MCL) on both sides.

**Fig. 6:**
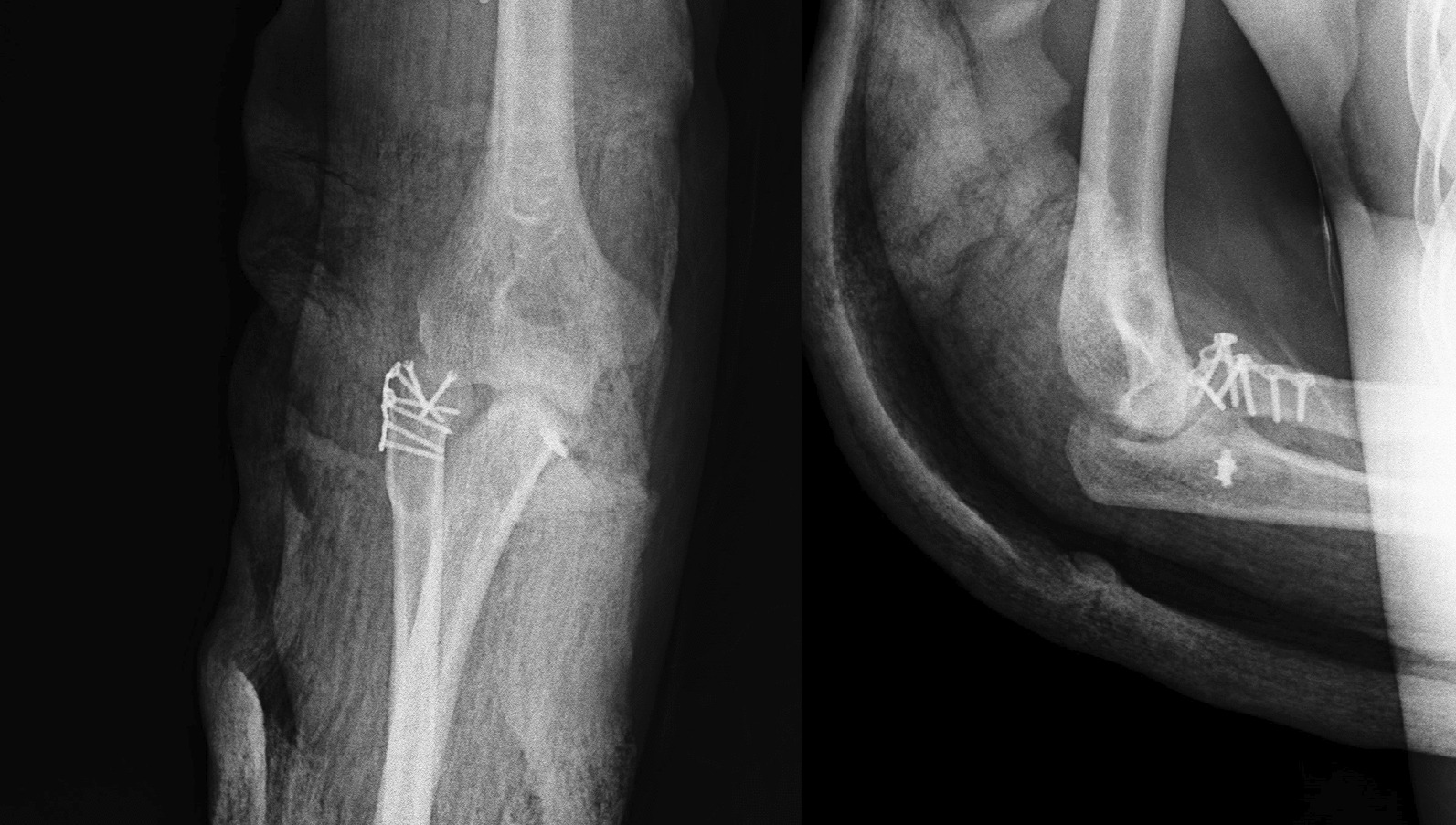
Postoperative right elbow X-rays

**Fig. 7:**
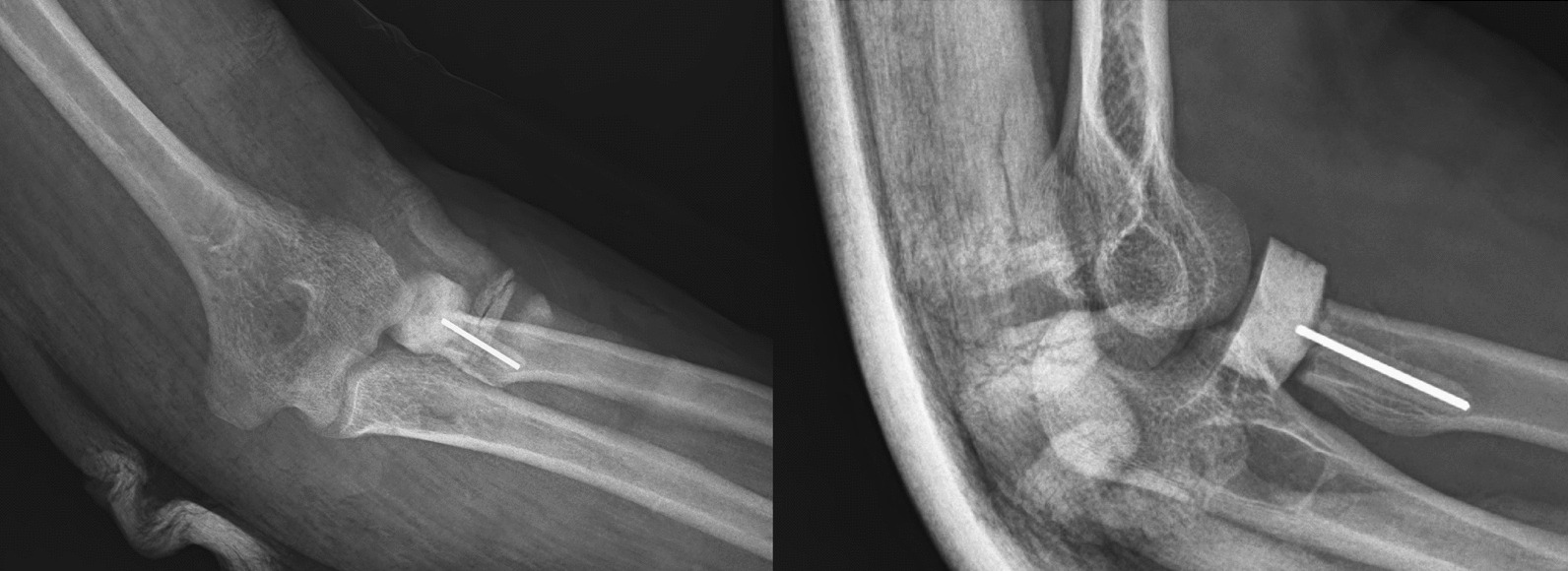
Postoperative left elbow X-rays

At the end, the surgical field was irrigated, and the fascia, subcutaneous tissue, and skin were sutured layer by layer, dressing was applied, and the splint was placed in pronation and 90 degrees of elbow flexion (Figs. 6,7).

Following a course of antibiotic therapy and wound care, the patient was discharged from the orthopedics and psychiatric wards following a 5-day postoperative period.

### Patient follow‑up and outcomes

Despite significant emphasis on the importance of precise follow-up after discharge, the patient failed to attend the follow-up visits at our facility to assess the wound and incision healing process. Attempts to establish communication with patient or his family via the telephone numbers listed in his medical record were unsuccessful.

Approximately 6 months after the trauma, patient was admitted to the psychiatric ward as a result of exhibiting psychotic symptoms caused by the use of psychoactive substances. Orthopedic consultation was sought to examine the patient regarding previous elbow surgeries and limited elbow range of motion (ROM).

According to the patient’s statements, the splints remained for about 3 weeks following surgery and no wound care was administered, after this period and with pain relief, he removed the splints and stitches himself using a knife.

**Fig. 8:**
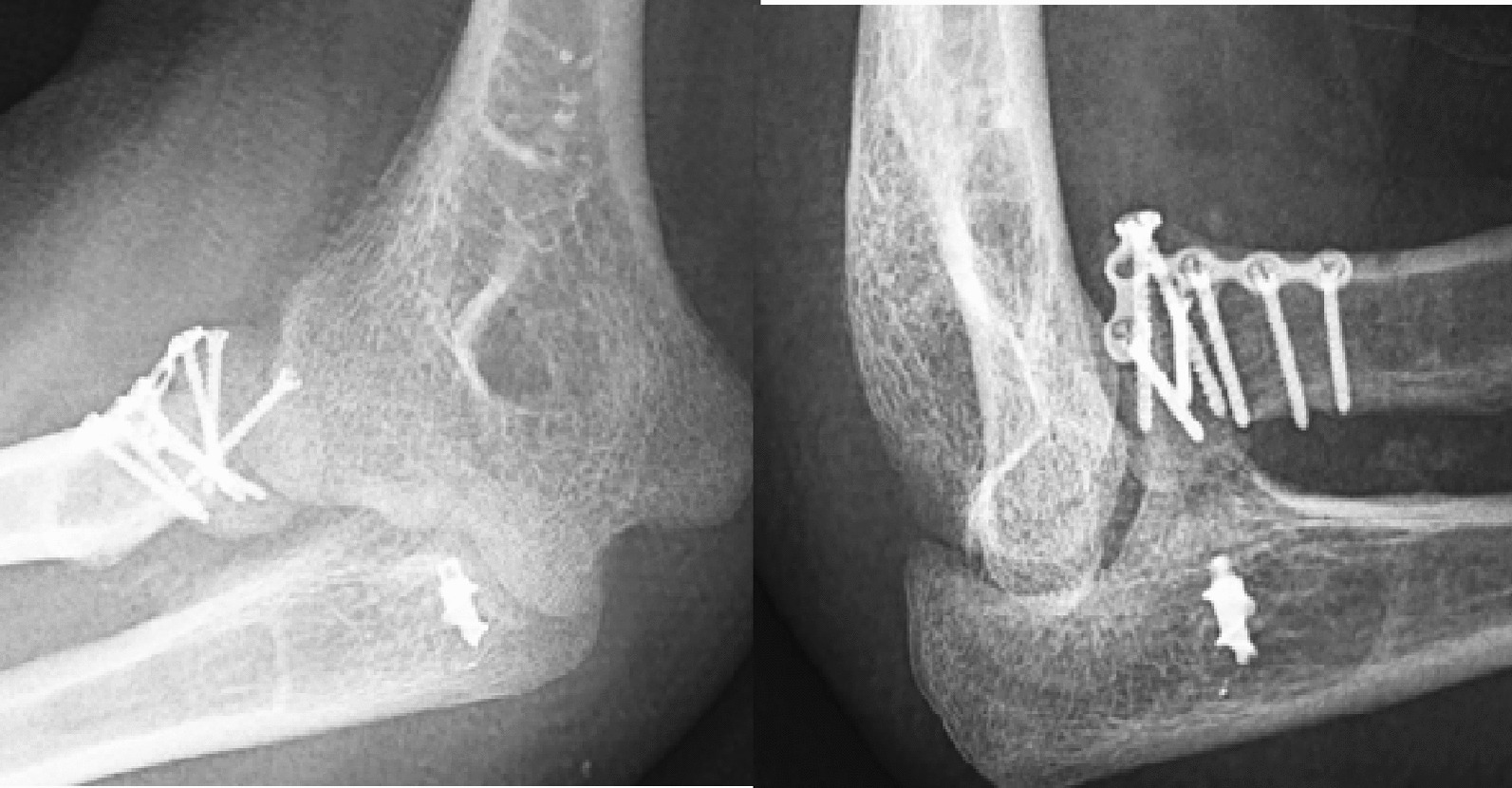
Radiographic images of right elbow in follow-up month 6; union was observed in radial head fracture

**Fig. 9:**
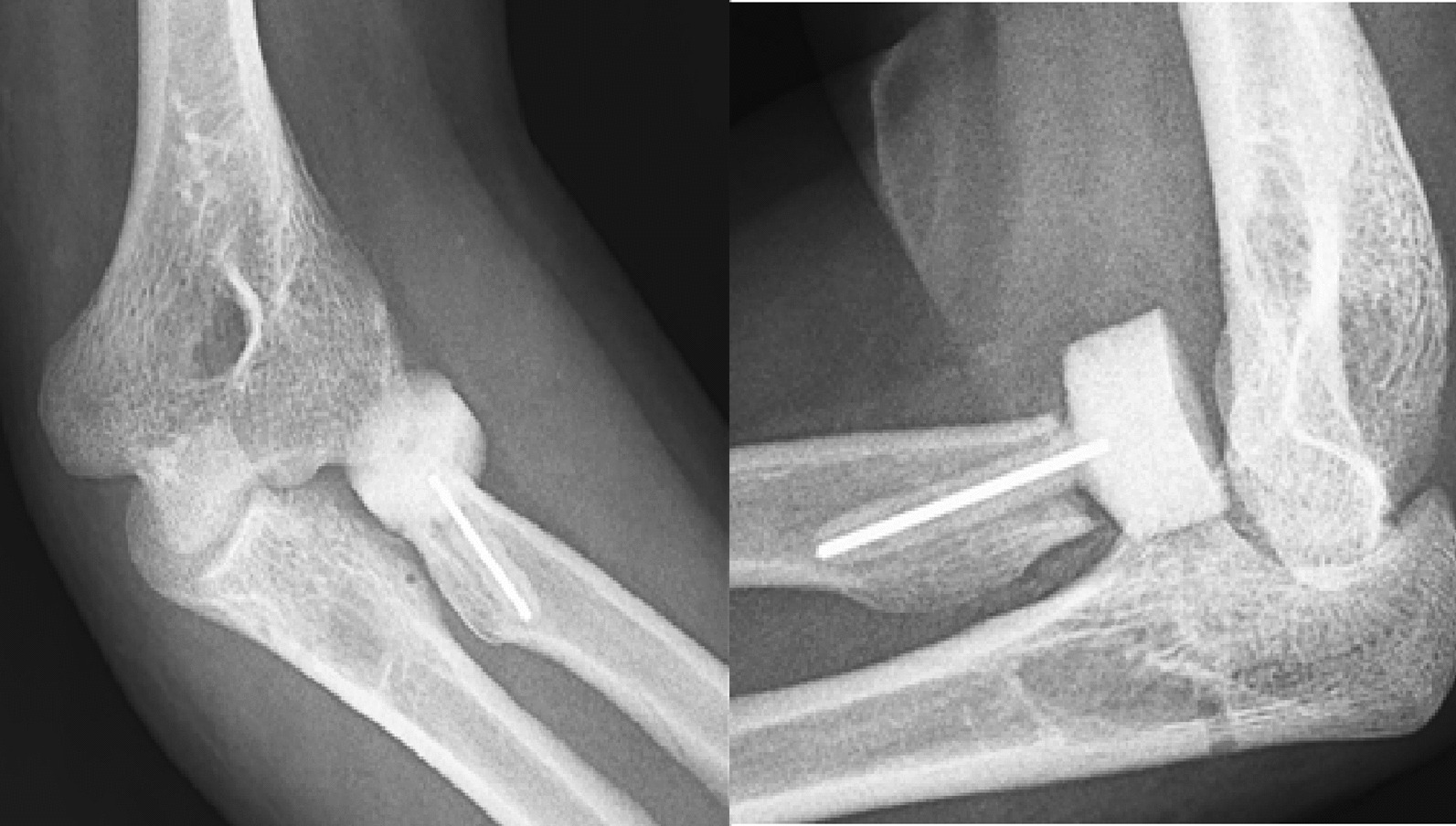
Radiographic images of left elbow in follow-up month 6; the joint is reduced and congruent, and no indications of spacer impairment or osteolysis in the proximal radius have been observed

The plain radiography revealed congruent elbows without any apparent evidence of subluxation. Evidence of union was detected in the RH fracture on the right side, and no pathological observations were noted in the X-rays of the left elbow (Figs. 8,9).

**Fig. 10:**
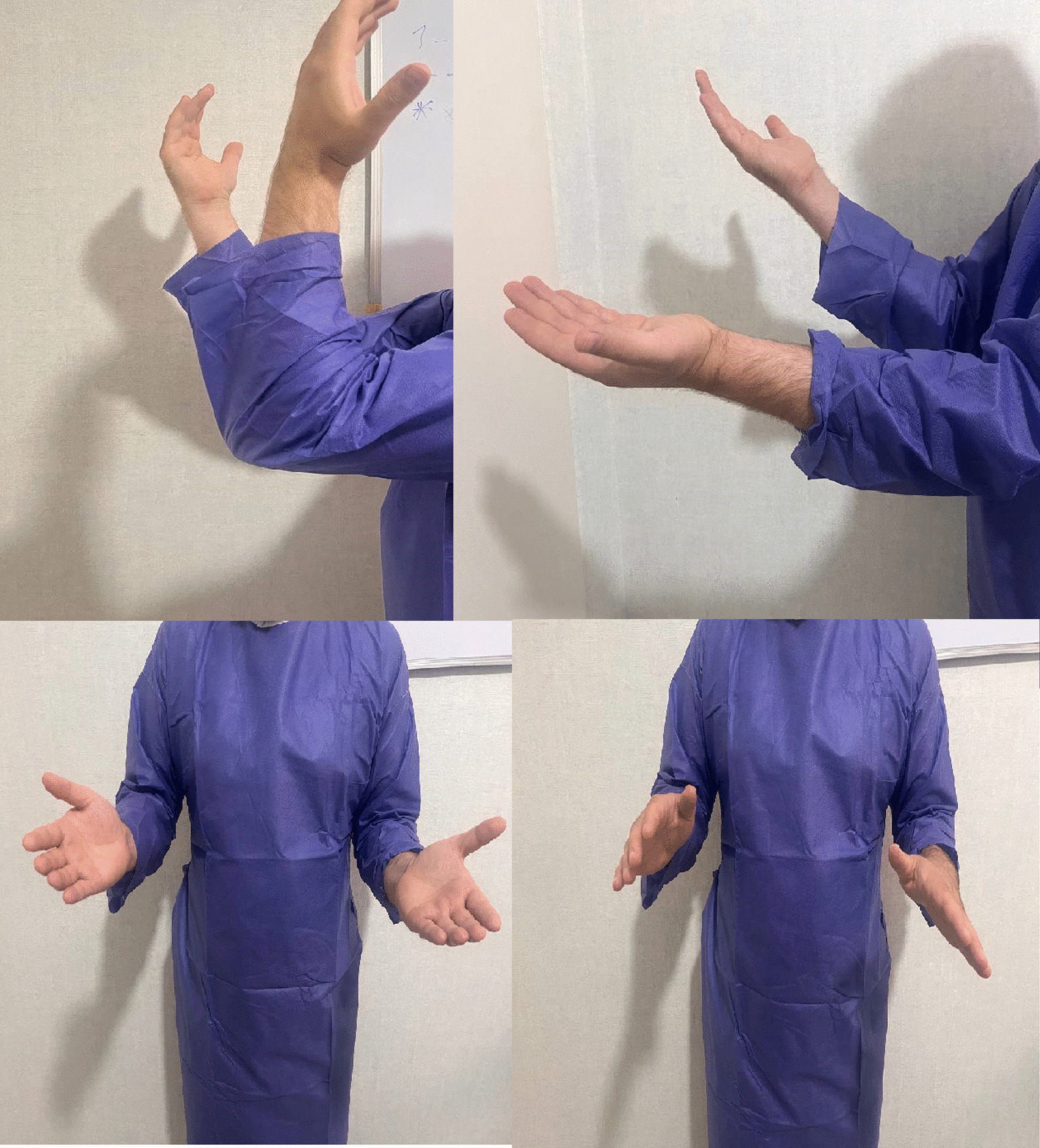
Restricted range of motion during follow-up month 6

During the patient examination, a notable improvement was observed in the skin condition of the elbow. The patient did not report any pain. However, the ROM of both elbows was significantly limited (Fig. 10).

In this case, despite the improvement of the elbows’ dermatological condition and becoming suitable for arthroplasty using a metallic prosthesis, the patient’s non-adherence, lack of cooperation, and psychological instability served as contraindications for this intervention. Conversely, concerning joint stability, it is well established in literature that a rigid but stable elbow is more effective than a flexible but unstable elbow. Therefore, it was determined by the care providing team that the spacer located in place of the left RH would be preserved until the substantial improvement of the patient’s psychological condition.

## Discussion

TTI is made up of a complex of ligamentous and osseous injuries of the elbow that are essential to the stability of the joint. Therefore, repairing these injuries are imperative to achieving joint stabilization. The injuries to bones in TTI include fractures of the RH and coronoid process, whereas the ligament injuries encompass ruptures of the LCL, anterior capsule, and MCL. With the exception of specific cases, the vast majority of injuries classified as TTI necessitate surgical intervention to maintain joint stability. The primary objective of such procedures is to expedite the restoration of joint stability and minimize the risk of stiffness [7].

Following the reduction of the joint dislocation, precise evaluation of potential bone injuries by 3D-CT scan is critical for establishing an effective treatment strategy. Coronoid fractures that occur in the context of TTI are frequently classified as Regan–Morrey type one or two, resulting in a relatively diminutive fragment size. In cases in which the coronoid fracture could not be fixed due to its small size or comminution, at the same time of repairing the anterior capsule of the joint the sutures can be passed around the fractured fragments, and they can be fixed using an anchor suture or a bone tunnel [8, 9], such as what we did in both elbows of this patient.

Given the significant role of the radiocapitellar joint in maintaining elbow stability, particularly in resisting valgus force, resection of the RH in TTIs where other joint stabilizers are also compromised may result in considerable residual instability within the joint [10]. Therefore, in cases in which the RH fracture is not reconstructable, the radiocapitellar joint should be replaced via hemiarthroplasty. To date, various studies have been published about different types of RH prostheses, highlighting the advantages and disadvantages of each. The available evidence suggests that the majority of RH arthroplasties exhibit satisfactory and similar mid-term longevity. However, controversies exist on the different long-term durability outcomes of various devices [11].

Capomassi *et al*. conducted a study on 38 patients requiring arthroplasty of the RH who underwent spacer arthroplasty due to limited access to RH prosthesis. Over the course of the average 53-month follow-up period, the outcomes were documented as follows: 14 patients classified as excellent, 14 patients classified as good, 8 patients classified as fair, and 2 patients classified as poor. Although radiological alterations were detected in 90% of the subjects, characterized by osteolysis of the proximal metaphysis of the radius, arthrosis of the capitulum, and heterotopic calcification, no significant correlation was found between these changes and the patient’s pain or limited ROM. The prevalent observation subsequent to surgical intervention in these individuals was the occurrence of crepitus during flexion and extension of the elbow, which was evidently unrelated to the patient’s nociceptive experience [12].

Clembosky *et al*. conducted a similar study to the previous publication involving 21 patients, wherein a similar surgical technique was employed. The investigators of that study were unable to access a RH prosthesis. Despite this limitation, their study yielded acceptable results, as evidenced by a mean follow-up period of 56 months. Within the cohort of 21 patients, the outcomes were deemed to be excellent for 9 individuals, good for 7 individuals, and fair for 5 individuals. The removal of prosthesis was undertaken in four patients with the aim of reducing their symptoms. These symptoms were primarily associated with overstuffing caused by the considerable dimensions of the spacer [13].

On the basis of the aforementioned studies, the utilization of a polymethylmethacrylate (PMMA) spacer as a substitute to metallic prostheses does not yield any adverse effects in the mid-term. Hence, the implementation of antibiotic-impregnated spacer arthroplasty has the capability to decrease the microbial load and maintain the stability of the elbow joint until the optimal time for definitive surgical intervention. This approach offers several advantages, including its affordability, availability, adaptability, and simplicity of extraction during the definite procedure [3].

To the best of our knowledge, no such therapeutic approach has been employed for this particular case in the current body of literature. Ultimately, this straightforward approach has the potential to prevent further complications in complex cases of TTI.

## Conclusion

In acute TTIs with unreconstructable RH, where the conditions for performing arthroplasty with metallic prostheses may not be suitable due to several factors, including contaminated wounds, unstable psychiatric condition, and low cooperation of the patient, temporary orthopedic cement spacers can be used as a viable alternative to maintain elbow biomechanics, stability, and sterility.

## Data Availability

The material presented in this study are available from the corresponding author on reasonable request.

## Abbreviations

TTI: Terrible triad injury
RH: Radial head
LCL: Lateral collateral ligaments
MCL: Medial collateral ligaments
3D-CT scan: Three-dimensional computed tomography scan
ROM: Range of motion
